# Comparative Analysis of Nutritional Quality, Serum Biochemical Indices, and Visceral Peritoneum of Grass Carp (*Ctenopharyngodon idellus*) Fed with Two Distinct Aquaculture Systems

**DOI:** 10.3390/foods13081248

**Published:** 2024-04-19

**Authors:** Rui Feng, Di Feng, Lingran Wang, Lan Zhang, Chang Liu, Fangran Ma, Meng Zhang, Miao Yu, Hongxia Jiang, Zhigang Qiao, Ronghua Lu, Lei Wang

**Affiliations:** 1 College of Fisheries, Engineering Technology Research Center of Henan Province for Aquatic Animal Cultivation, Engineering Lab of Henan Province for Aquatic Animal Disease Control, Henan Normal University, Xinxiang 453007, China; 18836225922@163.com (R.F.); f3187308@outlook.com (D.F.); wanglr0910@163.com (L.W.); zhanglan_1216@163.com (L.Z.); 18260381846@163.com (C.L.); mafangran@163.com (F.M.); mzhangshou@163.com (M.Z.); miaoyu@htu.edu.cn (M.Y.); jianghongxia2007@126.com (H.J.); 13503800008@126.com (Z.Q.); laoaiyika@aliyun.com (R.L.); 2 Observation and Research Station on Water Ecosystem in Danjiangkou Reservoir of Henan Province, Nanyang 474450, China

**Keywords:** *Ctenopharyngodon idellus*, pond intercropping, in-pond raceway system, serum biochemistry, visceral peritoneum, muscle quality

## Abstract

This study scrutinized the nutritional quality and serum biochemical indices of grass carp (*Ctenopharyngodon idellus*) cultivated in traditional pond intercropping (TPI) and in-pond raceway system (IPRS) aquaculture setups. The findings showed that the TPI group exhibited a superior water-holding capacity, while the IPRS showcased heightened crude lipid content and levels of textural properties such as springiness. Moreover, significant differences emerged in the fatty acid profiles, with the TPI group manifesting higher total polyunsaturated fatty acids (ΣPUFAs), EPA, DHA, and Σn-3, while the IPRS group exhibited elevated total saturated fatty acids (ΣSFAs). In terms of amino acids, valine and histidine levels were notably higher in the IPRS group, whereas lysine levels were reduced. Volatile compound analysis revealed significant variations, with the IPRS group containing more volatile substances with a better aroma, resulting in a better odor. The IPRS group performed better in serum biochemistry analysis. Additionally, grass carp in the IPRS group displayed an improved structure and greater coverage area of the visceral peritoneum, appearing lighter in color compared to the TPI group. TPI mainly influences nutritional elements; IPRSs primarily affect muscle texture, serum biochemistry, and overall health. This study aims to fill the gap in quality comparison research and provide an important scientific basis.

## 1. Introduction

In recent years, the global aquaculture industry has developed rapidly, ushering in huge opportunities and expanding on a large scale globally [1]. In the last seven decades, there has been a significant increase in the combined production of worldwide fishing and fish farming, rising from 19 million tons in 1950 to around 179 million tons in 2020, showing an average annual growth rate of 3.3%. According to FAO data, the annual average total production of global fisheries and aquaculture from 2015 to 2020 was 123 million tons. Observing the trend over the past decade, the global aquaculture output has gradually increased, with the cultivation, sales, and export of aquatic products becoming an essential component of economic development [2]. However, the expansion of the aquaculture industry inevitably comes at the expense of environmental degradation [3]. Aquaculture produces a significant amount of waste, including feces, and causes changes in water quality and the environment where aquaculture takes place [4,5]. Conversely, the decline of the water environment will impact the overall quality of aquatic goods [6]. Hence, the expansion of the aquaculture sector urgently necessitates the adoption of green and sustainable aquaculture modes [7]. It is essential for these methods to satisfy the need for top-notch aquatic goods without harming the water environment in aquaculture operations. With advancements in aquaculture technology, new green aquaculture modes have emerged, such as recirculating aquaculture systems, land-based push-water container cultivation, fish vegetable symbiotic culture mode, traditional pond intercropping, and in-pond raceway systems. Each aquaculture mode has its advantages and disadvantages, and the potential within them requires further exploration and utilization, as well as in-depth research on and comparisons of the impacts of different aquaculture modes on aquaculture species.

Food quality has become a major concern in recent years, as consumers are increasingly seeking products with higher nutritional value. Fish, being an important provider of proteins, minerals, vitamins, and essential fatty acids, has captured the interest and preference of individuals globally. The quality of fish can vary due to several factors, including odor, texture, nutritional content, temperature, and shelf life [8,9]. The quality of fish determines its economic value, and studies have shown that fish quality can vary with different aquaculture environments, primarily reflecting changes in morphological characteristics and flesh flavor [10]. Extensive studies have been carried out to investigate how different aquaculture methods affect the quality of fish, with trials conducted on a range of species like Yellow River carp (*Cyprinus carpio haematopterus*) and largemouth bass (*Micropterus salmoides*) [11,12,13,14]. However, with rapid advancements in global aquaculture technology and the emergence of new cultivation models, alongside increasingly stringent demands for fish meat quality, studying the impact of different cultivation modes on fish quality is of significant importance.

Grass carp (*Ctenopharyngodon idellus*), originating from China, is a freshwater fish species that has been introduced to numerous countries and regions, holding significant economic value and a long history of cultivation [15]. Consumers highly prefer grass carp due to its delectable taste, abundant nutrients, and delightful aroma, making it a significant economic fish species. Various methods are used for cultivating it, such as pond culture, polyculture, freshwater pond culture, cage culture, reservoir culture, paddy field culture, and traditional integrated breeding and farming modes. Among these, traditional pond culture is predominant, yet it tends to reduce the quality, texture, and market value of the fish flesh, leading to a muddy taste and soft muscle [16]. Additionally, the quality of traditional pond culture is hard to guarantee due to poor infrastructure and severe environmental pollution [17]. Consequently, the aquaculture industry is actively exploring and attempting more optimized cultivation modes and directions, such as grass carp traditional pond intercropping (TPI) and in-pond raceway systems (IPRS). Grass carp intercropping is not a mixed culture, but refers to the cultivation of a certain fish (not grass carp) as the core culture object, with grass carp and other fish of different sizes, species, and ages as additional culture objects, through the principle of complementary symbiosis, cultivating in the same pond, making full use of pond resources, and improving fish production. IPRSs represent a more innovative model that encourages fish movement, significantly improving fish flesh quality and texture [7]. Multiple studies have shown the advantages of IPRSs in improving different aspects of fish performance, with a focus on largemouth bass, common carp (*Cyprinus carpio*), and Nile tilapia (*Oreochromis niloticus*). Research has been carried out on the nutritional value of grass carp in various aquaculture settings [18], but there is a lack of comparative studies on different aspects of grass carp raised in traditional pond intercropping and in-pond raceway systems. This study aims to fill this research gap by comparing the morphological characteristics, muscle properties, nutritional components, volatile substances, serum biochemical indicators, and visceral peritoneum of grass carp under these two different aquaculture modes, providing a robust reference for regulating grass carp quality within IPRS and TPI aquaculture modes.

## 2. Materials and Methods

### 2.1. Experimental Materials

The grass carp used in this experiment were cultured from May 2022, when they weighed about 150 g, and then began to be cultured in both aquaculture systems until July 2023, which is 14 months of culture time, all raised at a specific aquaculture facility in Xinxiang. This farm utilizes groundwater for aquaculture, with the fish ponds having a depth of approximately 1.5 m, surrounded by concrete slopes, a mud-based bottom structure, and a cultivation density of about 30 fish per cubic meter. Following this, the grass carp in the IPRS were cultivated by Xinxiang Gaoketianyuan Agricultural Development Co., Ltd. The IPRS also used groundwater for aquaculture, with each raceway being 25 m long, 4.8 m wide, and covering an area of about 110 square meters. During the experiment, the IPRS group was fed the same type of feed, specifically Tongwei expanded feed. The TPI also used groundwater for aquaculture, with this group of grass carp consuming only natural forage within the pond, including grass. Intercropping is not mixed cultivation but refers to the method of raising a certain number of the same or different species of fry in the growth pond, feeding only the primary cultured species, while not feeding the other intercropped fish, with a density of about 0.05 fish per cubic meter. The TPI group obtained grass carp from ponds where carp were the primary species being raised, with grass carp, silver carp (*Hypophthalmichthys molitrix*), and bighead carp (*Aristichthys nobilis*) as additional species being raised. One hundred grass carp with identical growth periods were chosen from both the TPI and IPRS for study, with average weights of 1.24 ± 0.21 kg and 1.12 ± 0.20 kg, respectively.

### 2.2. Experimental Methods

#### 2.2.1. Determination of Morphological Characteristics of Grass Carp

Fifty grass carp were taken from each of the two aquaculture systems, and their body length and weight were measured. Among them, 22 fish were selected from each group for subsequent experiments. After 30 min of anesthesia with MS-222, their viscera and liver were weighed and measured to calculate the ratio to body weight. The following are the included formulas:

Condition factor (%) = 100 × (body weight/standard length^3^)

Hepatic steatosis index (%) = 100 × (liver weight/wet weight)

Visceral body ratio (%) = 100 × (visceral weight/wet weight)

#### 2.2.2. Determination of Physical Properties of the Back Muscle of Grass Carp

Muscle physical properties include muscle water-holding capacity, textural properties, and muscle fiber characteristics. All the muscles used were white muscle from the back of grass carp to remove the skin.

Water-holding capacity is related to parameters such as drip loss, centrifugal loss, liquid loss, stored loss, frozen leakage, and cooking loss. Jia’s calculation formula and method are used to determine water-holding capacity [11]. Samples of grass carp back muscle weighing 5 g were collected under the two aquaculture conditions for recording, and the six indexes mentioned above were then measured in order. In each group, 3 tails of grass carp were taken, and 3 parallels of each tail were taken to measure.

The grass carp back muscles were dissected into small 1.0 cm × 1.0 cm × 1.0 cm pieces for the precise evaluation of texture characteristics, including hardness, chewiness, cohesiveness, springiness, resilience, and gumminess. The muscle samples were measured after being cooked and cooled for 5 min, as well as when raw, using identical equipment. The data were then analyzed using the same processing method. Each group took 6 fish, with 3 parallels taken from each fish.

Muscle fiber characteristics were assessed by selecting three fish randomly (with three replicates per fish) from each of the two culture methods. The dorsal muscles were removed, cut into small 0.5 cm^3^ cubes, placed in a 4% tissue cell fixative (P1110, Solarbio, Corp., Beijing, China), dehydrated in wax, embedded, and sectioned. The muscle fibers were then dehydrated, embedded, and sectioned, the structural properties of the muscle fibers were observed using a vertical light microscope, and the images were captured and analyzed. The muscle fibers of grass carp were measured and analyzed for their structure, diameter, and density using the Caseviewer software (version 2.4).

#### 2.2.3. Determination of Nutrient Components of the Back Muscle of Grass Carp

Muscle nutrition in grass carp is influenced by traditional muscle components and levels of fatty acids and amino acids. To identify the traditional muscle components, three experimental fish were randomly chosen from each group for analysis of traditional muscle composition, with their dorsal muscles used for the analysis and three measurements taken from each group. The methods of measurement and standards used for each nutrient component were as follows.

Moisture content was determined using the direct drying method at 105 °C. The muscle samples were dried at 105 °C for 4 h in a drying oven, cooled for 30 min, dried for 1 h, and then weighed. We measured the difference between the original mass and the dried mass of the muscle sample and calculated the moisture content (the weighing bottle was dried to a constant weight to reduce the impact of the weighing bottle’s mass on the results). The ash content was determined using the volatile constant weight method in a muffle furnace. An appropriate amount of muscle sample was placed in the crucible, carbonized to smokeless on the electric furnace, and then burned in the muffle furnace at 550 ± 20 °C for 4 h, taken out when the temperature of muffle furnace dropped below 200 °C, cooled in air for 1 min, and put into a dryer for 30 min to weigh. Then, we calculated the ash content. The crude lipid content was determined using the Soxhlet extraction method. An appropriate amount of the sample was packed with filter paper and immersed in petroleum ether solution. It was taken out after being measured by a fat tester for 1.5 h. It was dried in an oven at 105 °C for 4 h, and then taken out and weighed. The difference between the before and after mass was calculated to calculate the crude lipid content. The crude protein content was determined using the micro Kjeldahl method. After a proper amount of muscle sample was digested in a digestion tube with a catalyst, it was titrated by a hydrochloric acid solution after being treated by the Kjeldahl nitrogen determination method, and the color changed to grayish red as the end point. Then, we calculated the crude protein content.

The fatty acid makeup of grass carp muscle was analyzed using a 7890B gas chromatograph (GC-MS 7890B, Agilent, Corp., Santa Clara, CA, USA), and the amounts of fatty acids in each section were calculated using the area normalization method. After the muscle samples were first hydrolyzed by acid hydrolysis, the fat was extracted using a mixed solution of ether and petroleum ether to obtain fat extract. After that, the saponification reaction of fat and methyl esterification of fatty acid were carried out. The specific process was that the fat extract was first saponified with sodium hydroxide solution to produce fatty acid sodium salt, and then esterified with methanol to produce fatty acid methyl ester. The fatty acid methyl ester content was quantified by capillary column gas chromatography based on the content of various fatty acid methyl esters and conversion coefficients to calculate the content of various fatty acids. 

The muscle amino acid content was determined using the method outlined in the National Standard for Food Safety. Samples were processed and analyzed with an amino acid autoanalyzer A300, excluding tryptophan due to acid hydrolysis destruction. The proportion of each primary amino acid was determined using the area normalization technique.

#### 2.2.4. Determination of Volatile Substances in the Back Muscles of Grass Carp

The procedure for identifying volatile compounds in grass carp muscle involved utilizing a FlavourSpec^®^ flavor analyzer with the gas chromatography–ion mobility spectrometry (GC-IMS) technique. To determine the sample, 2 g was weighed and then transferred into a 20-milliliter vial for headspace analysis. Incubation lasted for 15 min at a temperature of 80 degrees Celsius, with an injection volume of 500 microliters. The GC-IMS setup included the use of an MXT-5 column measuring 15.00 m × 0.53 mm with a thickness of 1.00 μm. The injection needle was set at a temperature of 85 °C, while the column temperature was maintained at 60 °C. The carrier gas/drift gas utilized was N_2_ with a purity of 99.999%. The carrier gas flow rate conditions involved starting with a flow rate of 2 mL/min for 2 min, followed by an increase to 10 mL/min for 8 min and then a further increase to 100 mL/min for 10 min, resulting in a total analytical time of 20 min. Examination of IMS conditions was conducted at a temperature of 45 degrees Celsius, with a drift gas flow rate of 150 milliliters per minute. The assay utilized various analytical software such as Laboratory Analysis Viewer 0.4.03, Reporter plug-in, Gallery Plot plug-in, Dynamic PCA plug-in, and GC × IMS Library Search. To ensure data accuracy, three parallel measurements were conducted for each sample.

#### 2.2.5. Analysis of Serum Biochemical Indices in Grass Carp

Nine grass carp in the experiment were chosen at random from the two different cultivation methods. After anesthesia with MS-222, an appropriate amount of blood was extracted (unclotted samples were used to reduce the error), placed in centrifuge tubes, and stored at 4 °C overnight. The samples were centrifuged to obtain serum, which was frozen and stored at −20 °C. Following that, the levels of total protein (TP), albumin (ALB), total cholesterol (TCHO), triglyceride (TG), glucose (GLC), alkaline phosphatase (ALP), lysozyme (LZM), alanine aminotransferase (ALT), and aspartate aminotransferase (AST) were measured using the Nanjing Jianjian Bioengineering Institute kit.

#### 2.2.6. Analysis of Visceral Peritoneum in Grass Carp

To compare the color of the visceral peritoneum across two aquaculture modes, this experiment employed the method of observing color plaques. Initial visual inspection revealed significant color differences between the two aquaculture methods. To corroborate these observations, a microscopic examination of the grass carp’s visceral peritoneum was conducted. Utilizing both visual inspection and microscopic examination, the present study observed the color plaques on the visceral peritoneum of grass carp reared under two distinct aquaculture modes. Furthermore, a comparative analysis was conducted to assess the size and color intensity of these color plaques.

### 2.3. Data Analysis

The data involved in the experiment are expressed in the form of mean ± standard deviation (mean ± SD); Microsoft Excel 2019 was used to organize the data, GraphPad Prism 6.02 was used to make graphs, and the SPSS software (released by IBM Corp. in 2013, IBM SPSS Statistics for Windows, version 23.0.) was used. A *t*-test was performed so as to determine whether there was a significant difference between the groups of data. At the 95% confidence interval, *p* < 0.01 indicates highly significant differences, *p* < 0.05 indicates significant differences, and *p* > 0.05 indicates insignificant differences.

## 3. Results

### 3.1. Morphological Characteristics

**Figure 1:**
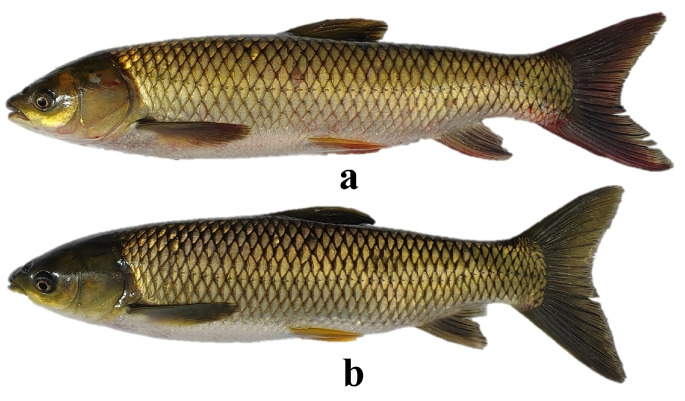
Comparative picture of pond intercropping and in-pond raceway system of *Ctenopharyngodon idellus*: (**a**) pond intercropping mode (TPI); (**b**) in-pond raceway system (IPRS).

Two sets of grass carp with similar body weights were chosen from two different aquaculture methods. In this instance, various morphological markers were contrasted, with the findings displayed in Table 1. The comparison findings indicated that grass carp raised in the IPRS exhibited notably greater fullness and visceral-to-body ratio compared to the TPI group (*p* < 0.05), s significantly higher liver-to-body ratio than the TPI group (*p* < 0.01), and a significantly shorter body length than the TPI group (*p* < 0.01). The results showed that grass carp raised in the IPRS had a more plump and symmetrical body shape, with a healthy and smooth surface; grass carp raised by TPI had a slenderer body shape and a reddish surface (Figure 1).

### 3.2. Physical Properties

#### 3.2.1. Muscle Water-Holding Capacity in Grass Carp

The six indicators of muscle water-holding capacity in the two aquaculture modes are shown in Table 2. Based on the information provided in Table 2, there were no notable variations in drip loss, centrifugal loss, stored loss, and frozen leakage between the two aquaculture methods for grass carp (*p* > 0.05). However, the TPI group exhibited significantly lower cooking loss compared to the IPRS group (*p* < 0.01), and the TPI group also had significantly lower liquid loss than the IPRS group (*p* < 0.05). Overall, the comprehensive data indicate that grass carp muscles in the TPI group possess superior water-holding capabilities and are more convenient for daily storage.

#### 3.2.2. Textural Properties

**Figure 2:**
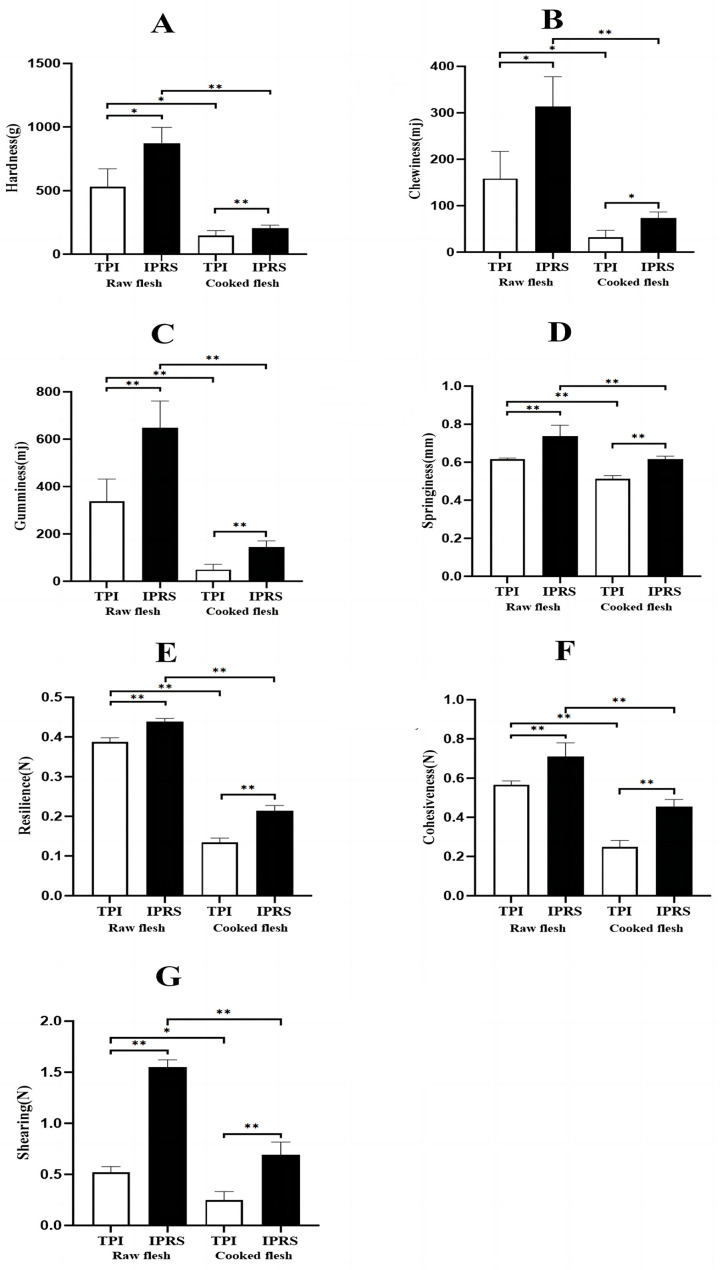
The raw and cooked muscle textural properties of *Ctenopharyngodon idellus* cultured in different aquaculture modes (*n* = 6). * indicates significant difference (*p* < 0.05); ** indicates extremely significant difference (*p* < 0.01). Raw-fillet and cooked-fillet texture of *Ctenopharyngodon idellus* cultured under different aquaculture modes: (**A**) hardness, (**B**) chewiness, (**C**) gumminess, (**D**) springiness, (**E**) resilience, (**F**) cohesiveness, and (**G**) shearing.

The textural properties of grass carp muscle in the two aquaculture modes are shown in Figure 2. The analysis of the data indicates that the hardness, chewiness, cohesiveness, springiness, resilience, gumminess, and shearing of grass carp muscle in the IPRS group were notably greater than in the TPI group (*p* < 0.05), while the hardness, chewiness, cohesiveness, springiness, resilience, gumminess, and shearing of cooked grass carp muscle in both groups were notably lower than in the uncooked flesh (*p* < 0.05). The findings indicated that the textural properties and muscle firmness of grass carp in the IPRS group surpassed those in the TPI group, with a superior taste compared to the TPI group.

#### 3.2.3. Characteristics of Muscle Fibers

**Figure 3:**
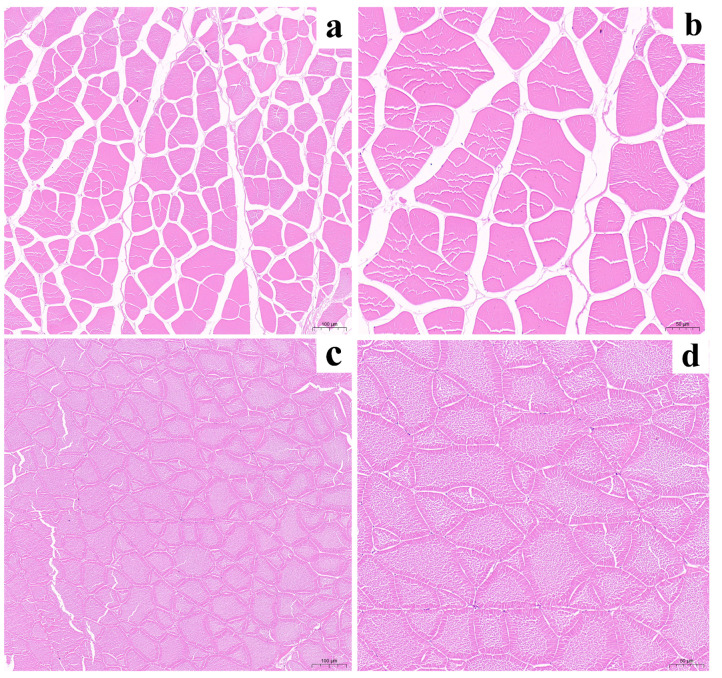
Muscle cross section of *Ctenopharyngodon idellus* in TPI and IPRS group: (**a**,**b**) 100 µm and 50 µm in TPI group; (**c**,**d**) 100 µm and 50 µm in IPRS group.

The Table 3 data show that the muscle fibers in the IPRS group were shorter and thinner compared to the TPI group, with a higher density of muscle fibers in the IPRS group than the TPI group (*p* < 0.01). The results indicate that the TPI group exhibited larger muscle fiber lengths and diameters with lower density and more connective tissue between muscle fibers. In contrast, the grass carp muscle fibers in the IPRS group were shorter and narrower, evenly spread out, and had reduced connective tissue and a higher density of muscle fibers (Figure 3).

### 3.3. Muscle Nutrients

#### 3.3.1. General Nutrients

Table 4 shows that the IPRS group had a notably lower ash content compared to the TPI group (*p* < 0.01), while the crude lipid content was significantly higher in the IPRS group than in the TPI group (*p* < 0.05). The water and crude protein contents did not show a notable variance between the two aquaculture methods for grass carp (*p* > 0.05). The findings indicated that the TPI group had less fat and more ash, while the IPRS group had more fat and less ash.

#### 3.3.2. Fatty Acid Composition Analysis

The analysis of the lipid composition in grass carp muscle from two different aquaculture methods revealed the presence of 18 fatty acids, consisting of four saturated fatty acids (ΣSFAs), six monounsaturated fatty acids (ΣMUFAs), and eight polyunsaturated fatty acids (ΣPUFAs). In both culturing modes, grass carp muscle had the highest C16:0 content, with saturated fatty acids making up over 35% of the total fatty acid content. There was no significant difference in the monounsaturated fatty acid content. Additional findings can be found in Table 5. The grass carp muscle in the IPRS group had a significantly higher ΣSFA content compared to the TPI group (*p* < 0.01), while the levels of ΣPUFAs, EPA, and DHA were significantly lower in the IPRS group than in the TPI group (*p* < 0.01). The levels of Σn-6 and Σn-9 were similar in both groups; however, the IPRS group had significantly lower levels of Σn-3 compared to the TPI group (*p* < 0.01). These findings indicate that the IPRS group had elevated levels of saturated fatty acids, while the TPI group had higher levels of beneficial unsaturated fatty acids like EPA, DHA, and Σn-3. Upon examination of the properties and roles of various fatty acids, it was found that the TPI group contained a greater amount of muscle-friendly, premium fatty acids and superior nutritional value.

#### 3.3.3. Amino Acid Composition Analysis

Muscle samples from both groups of grass carp were analyzed for seventeen amino acids, with nine being essential (EAAs), four non-essential (NEAAs), and four considered umami (UAAs), excluding tryptophan. Table 6 indicates that there were significant differences in the total levels of valine, lysine, histidine, and tasty amino acids between the two groups (*p* < 0.05). Valine and histidine levels were notably higher in the IPRS group compared to the TPI group (*p* < 0.05), while lysine and ΣUAA levels were significantly lower in the IPRS group (*p* < 0.05). Nonetheless, the EAA and NEAA groups did not show a notable distinction (*p* > 0.05). The results indicate that the two culture modes, TPI and IPRS, did not affect the amino acid types of grass carp muscle, but affected the contents of valine, lysine, histidine, and umami amino acids. Among them, the TPI group had a higher content of umami amino acids, and the overall amino acid levels did not differ significantly.

### 3.4. Analysis of Volatile Substances in Muscles

**Figure 4:**
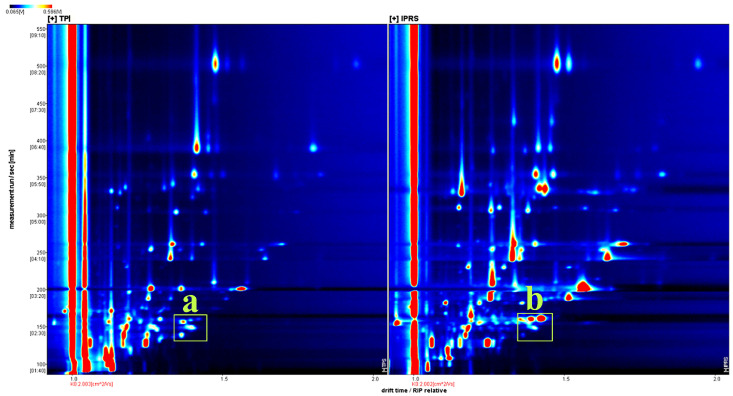
Gas-phase ion mobility spectrum of *Ctenopharyngodon idellus* muscle in two aquaculture modes. The ordinate represents the retention time (s) of gas chromatography, and the abscissa represents the ion migration time (normalized). The points on both sides of the reaction peak represent volatile organic compounds. Colors indicate the concentration of the substance, with white representing a low concentration and red representing a high concentration. The deeper the color, the higher the concentration. Its color represents the concentration of the substance, with darker colors indicating greater concentrations, white indicating lower concentrations (e.g., a), and red indicating higher concentrations (e.g., b).

**Figure 5:**
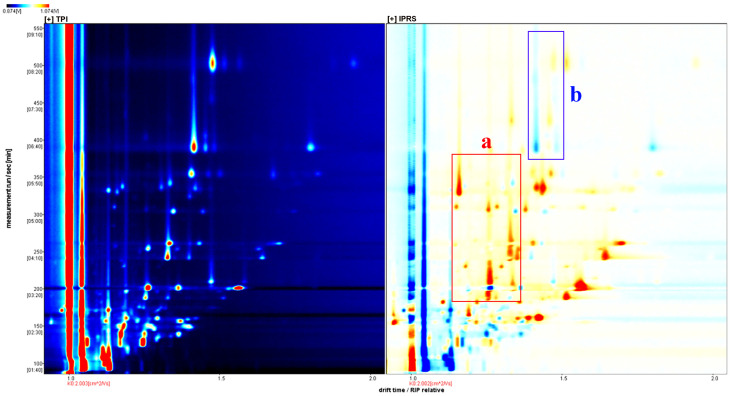
Differential gas-phase ion migration spectra of volatile substances in *Ctenopharyngodon idellus* muscle in two aquaculture modes. Red indicates a higher concentration of the substance in the sample than the reference sample (a), while blue indicates a lower concentration (b). The darker the red, the more the concentration of the corresponding substance is higher than that of CHG. The darker the blue, the reverse is true.

**Figure 6:**
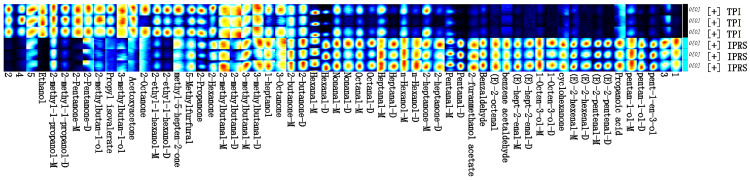
Fingerprint analysis of volatile substances in *Ctenopharyngodon idellus* muscle in two aquaculture modes. -M and -D, which are the monomers and dimers of the same substance, are presented behind some substances, and the numbers refer to unidentified peaks.

Figure 4, Figure 5, Figure 6 and Figure S1 display the GC-IMS spectra of volatile flavors found in grass carp muscle under various aquaculture conditions. The volatile flavors were analyzed using a FlavourSpec^®^ flavor analyzer for grass carp muscle in the two aquaculture modes, and the three-dimensional GC-IMS spectra were derived. The migration time, retention time, and signal peak intensity are represented by the three axes (X, Y, and Z, respectively) and can be used to visualize the variations in volatile organic compounds across different modes. However, the top view was selected for comparison in this study for ease of observation. A two-dimensional top view was created by the Reporter plug-in (Figure 4). In Figure 4, there is a red vertical line located at the horizontal coordinate 1.0. The RIP peak of Figure 4 (reactive ion peak, normalized) visually indicates the variations in component and concentration among different grass carp muscles based on the presence or absence of peaks (color dots) and the intensity of color. The y axis indicates the gas chromatogram’s retention time in seconds, while the x axis shows the normalized ion migration time. Every point surrounding the RIP peak signifies a volatile organic compound. The substance’s concentration is shown by its color, with darker shades representing higher concentrations, white representing lower concentrations, and red representing higher concentrations. The gas-phase ion mobility spectra of grass carp muscle varied significantly between the two aquaculture modes, as depicted in the comparative plots below with distinct differences in the a and b regions of Figure 4.

To better understand and contrast the variations in volatile elements in fish, the grass carp samples in TPI mode were chosen as a baseline for comparison. The spectra of the IPRS samples were then subtracted from the baseline to create difference comparison graphs of various fish samples, depicted in the figure below (Figure 5). When the VOC contents in the target sample and the reference match, the background turns white after deduction. A red color indicates a higher concentration of the substance in the target sample compared to the reference, like in area a, while a blue color indicates a lower concentration in the target sample, as seen in area b. The difference between the two samples is shown in the following figure (Figure 5). The more intense the hue, the larger the variation in the concentration of this unstable compound in the two aquaculture methods.

To accurately identify the distinct chemical compound present in grass carp muscles under various aquaculture conditions, all peaks were chosen for comparison in the fingerprinting process (Figure 6). The figure displays signal peaks chosen in each sample in rows and the signal peaks of the same VOC in various samples in columns, providing a comprehensive view of the VOC information in each sample and the variations in VOCs across samples. The differences in volatile components between several groups of samples can be visualized very well from the following figure (Figure 6), in which ethanol, 2-methylpropanol, 2-methylbutanol, 2-ethylhexanol, acetone, 2-pentanone, 2-hexanone, 2-octanone, etc., were higher in TPI; glutaraldehyde, hexanal, heptanal, octanal, nonanaldehyde, (E)-2-pentenal, (E)-2-hexenal, (E)-2-heptenal, (E)-2-octenal, benzaldehyde, and benzene acetaldehyde were higher in IPRS, and the differences in VOC fractions between the two groups of samples were significant.

The volatile components in the fish were determined by qualitative analysis using GC-IMS, and the qualitative spectra are shown in Figure S1. A total of sixty volatile components were determined in the two samples (containing different forms of the same substance due to different concentrations), and five of them were not identified and classified due to the imperfections of the volatile substances database. Complete information on the remaining 37 VOCs is presented in Table S1. A one-way ANOVA revealed that the IPRS group demonstrated higher contents of key compounds such as nonanal, hexanal, heptanal, hexanol, 1-octen-3-ol, 2-heptanone, 2-butanone, 2-methylbutanal, and 3-methylbutanal (*p* < 0.05). Conversely, the TPI group exhibited elevated levels of ethanol and 2-ethylhexanol (*p* < 0.01). Complete information on the remaining 37 VOCs is presented in the Supplementary Materials. The IPRS group had a higher overall level of volatiles, which contained a variety of high levels of flavorful aldehydes, while the TPI group had prominent levels of ethanol and 2-ethylhexanol. The results indicated that the two aquaculture modes did not affect the types of muscle volatiles, but significantly affected the content of volatiles in the muscle. The IPRS group had a higher overall level of volatiles, which contained a variety of high levels of flavorful aldehydes, while the TPI group had prominent levels of ethanol and 2-ethylhexanol. After analyzing the characteristics of each type of volatile substance, it was determined that the grass carp muscles in both groups exhibited a certain clear flavor, but the muscles of the TPI group were accompanied by a strong earthy flavor, and the meat of the IPRS group had a richer and more pleasant odor.

### 3.5. Serum Biochemical Indices

Table 7 displays the findings of nine serum biochemical indices in grass carp from both aquaculture methods, with measurements taken. According to Table 7, the IPRS group showed significantly higher levels of TP, ALB, TG, and ALP compared to the TPI group (*p* < 0.01). Conversely, the concentrations of GLC, TCHO, ALT, and AST were significantly lower in the IPRS group than in the TPI group (*p* < 0.01), while LZM levels did not differ significantly between the two groups. The analyzed data could indicate that the different aquaculture modes had a very significant effect on the serum biochemical index contents, particularly focusing on the levels of TP, AST, and ALP.

### 3.6. Comparative Analysis of Grass Carp Visceral Peritoneum

**Figure 7:**
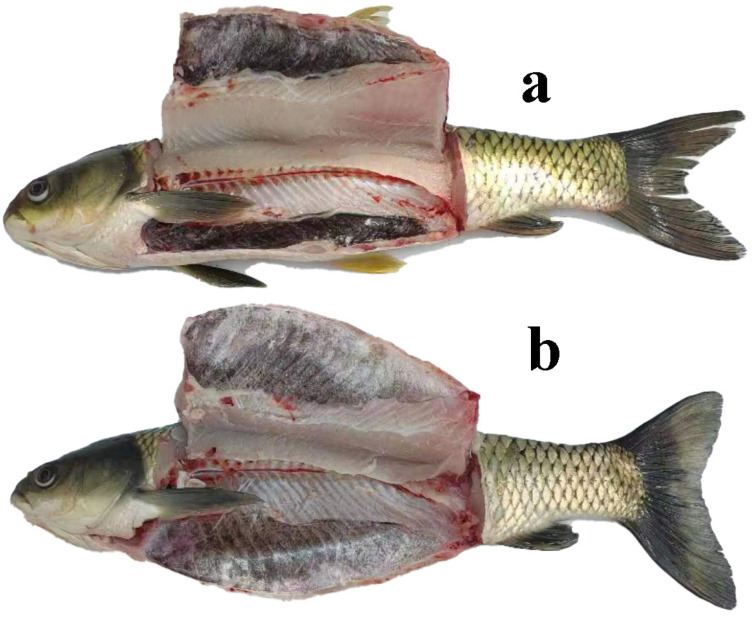
Comparative picture of *Ctenopharyngodon idellus* visceral peritoneum of traditional pond intercropping and in-pond raceway system: (**a**) traditional pond intercropping mode (TPI); (**b**) in-pond raceway system (IPRS).

Two groups of grass carp were dissected and the visceral peritonea were unfolded, and the results are shown below (Figure 7). The findings indicated that various aquaculture methods significantly impacted the visceral peritoneum, which was reflected in the morphology structure, pigmentation, and coverage of the visceral peritoneum. In the IPRS mode, the visceral peritoneum of grass carp showed a light, relatively uniform color, and no obvious black spots or plaques. However, under the mode of TPI, the visceral peritoneum of the grass carp was obviously dark, and the black areas were unevenly distributed; some parts were darker, and some parts were relatively light. The overall results show that the IPRS group had a significantly lighter color and higher coverage of the visceral peritoneum, and the morphology of the visceral peritoneum was more complete than that of the TPI group.

## 4. Discussion

### 4.1. Effects of Different Aquaculture Modes on the Physical Properties of Grass Carp Muscle

Muscles’ capacity to retain moisture under external conditions is known as water-holding capability (WHC), which is influenced by their unique chemical makeup and physical characteristics. It is a physicochemical index for evaluating muscle quality, significantly affecting muscle texture [19]. A higher WHC indicates a slower loss of nutrients and flavor substances, longer storage times, and better muscle quality. WHC can be measured by indicators such as drip loss, centrifugal loss, liquid loss, stored loss, frozen leakage, and cooking loss, all of which are inversely related to WHC [11]. The TPI group had notably reduced liquid loss compared to the IPRS group in the experimental findings (*p* < 0.05), with an even more significant decrease in cooking loss (*p* < 0.01) (Table 2). This indicates that the TPI group has a higher WHC, related to the connective tissue between muscle fibers. Studies have shown that an increase in connective tissue in muscles can enhance the ability to retain moisture and prevent juice loss [20]; when muscle is compressed or cooked and contracts, a certain amount of connective tissue reduces water mobility, retaining water within the muscle tissue [21]. The significantly lower WHC in the IPRS group is linked to its low connective tissue content, differing from the general assumption that high muscle fiber density correlates with high WHC, proving that WHC is also constrained by muscle connective tissue, forming a prerequisite for water retention that includes both muscle fiber density and connective tissue elements [22].

Muscle fiber properties are important physical indicators for evaluating meat quality, closely related to muscle meat quality. Muscle fiber properties are mainly influenced by diameter and density; smaller fiber diameters at constant volume lead to higher density, resulting in firmer muscle with better texture. This research revealed that the size of muscle fibers in the IPRS group (Table 3), both long and short diameters, were notably smaller compared to the TPI group (*p* < 0.01), while also showing a higher fiber density (*p* < 0.01). Research suggests that exercise training promotes fish growth by regulating muscle fiber hyperplasia, possibly due to enhanced movement in pond raceway systems promoting fiber proliferation and reducing inter-fiber cell spacing, leading to shorter fiber diameters and increased overall density [23,24].

Muscle fiber structure and texture properties are closely related, affecting the textural properties of fish meat [22]. The characteristics of fish flesh texture, which are crucial for evaluating the quality of food, encompass attributes like hardness, chewiness, cohesiveness, springiness, resilience, and gumminess, with hardness playing a key role in determining the quality of the meat [24,25,26]. The IPRS group in the grass carp raw flesh category exhibited notably elevated levels of hardness, chewiness, cohesiveness, springiness, resilience, gumminess, and shearing in comparison to the TPI group (*p* < 0.05) (Figure 2), consistent with the results reported by Zhang but with slightly different aquaculture modes [27]. The cooked flesh group showed similar results, with the IPRS group showing significantly higher values in the same parameters (*p* < 0.05); the texture property indicators of both groups of cooked meat were significantly lower than in the raw meat group (*p* < 0.05). This study demonstrates that smaller muscle fiber diameters and higher muscle density correlate with increased hardness and chewiness, thereby enhancing meat quality [26,28]. This suggests a positive correlation between muscle fiber density and indicators such as hardness and chewiness, similar to findings from Jia’s study [11]. Conversely, the lower fiber density and higher connective tissue content in the TPI group reduce texture properties such as hardness and springiness while inhibiting moisture loss, resulting in a softer texture and higher moisture content in TPI-group meat [21,25]. According to Luo’s result, reduced muscle hardness impacts meat quality, while improvements in hardness, chewiness, and springiness enhance meat quality, indicating that IPRS plays a positive role in improving muscle texture properties and meat quality [29].

### 4.2. Impact of Various Aquaculture Modes on the Nutritional Composition of Grass Carp Flesh

Muscle typically contains moisture, ash, crude protein, and crude lipid as its primary nutritional components, with protein and lipid being the most concentrated. The muscle quality is greatly influenced by the protein and lipid content. Excessive lipid in cultivated fish can negatively impact taste [30]. Our experimental findings indicate that there were no notable variances in moisture and crude protein levels in grass carp muscles between the two cultivation methods (Table 4). However, the ash content in the IPRS group was notably lower than in the TPI group (*p* < 0.01), while the crude lipid content was significantly higher in the IPRS group compared to the TPI group (*p* < 0.05). These findings are consistent with Gharti’s result, who reported increased crude lipid levels in grass carp raised in raceways, which is also in line with the results of Hai-Shan, where the crude lipid content in gibel carp (*Carassius auratus gibelio*) muscle significantly increased after swimming training [16,31]. The fundamental reason for the differences in nutritional content between aquaculture modes lies in the variation in food and environment [32]. Multiple research studies indicate that swimming activity at specific levels can boost the crude lipid content in fish muscles, as raceway cultivation promotes moderate exercise, enhancing muscle growth and indirectly leading to fat deposition. However, when the exercise exceeds a certain threshold, it leads to direct fat breakdown for energy, decreasing fat content and leading to reduced body weight and condition factor [33], consistent with the findings of Li, where fat levels initially rise, then decrease, and finally stabilize [34]. During TPI mode, grass carp consume grass and other natural food, leading to reduced muscle fat levels and increased moisture levels. The decreased fat levels are linked to the heightened activity and vigilance of grass carp in pond intercropping, as stated in Gharti’s study [16]. The higher fat content in the IPRS group leads to smoother muscle; if the fat content is lower, the muscle becomes coarser, reducing palatability [30]. The higher ash content in the TPI group is closely related to its living environment, where still-water ponds contain rich sediment and aquatic plants, providing abundant inorganic salts and minerals.

Fatty acids play a crucial role in evaluating the nutritional value of fish muscle, necessary for overall health, with their nutritional content mainly determined by the levels of Σn-3, Σn-6, EPA, and DHA. Aquaculture in raceway systems primarily enhances the quality of muscle nutrition by regulating muscle fiber texture and nutritional components through exercise [35,36,37,38]. The findings from the experiment indicated that the level of saturated fatty acids in the IPRS group was notably greater compared to the TPI group (*p* < 0.01) (Table 5). Additionally, the levels of PUFAs, EPA, DHA, and Σn-3 were significantly lower in the IPRS group (*p* < 0.01), which contradicted the prevailing belief in various studies like Zhu’s that moderate swimming can greatly boost the levels of PUFAs, EPA, and DHA in fish muscle [39]. This is also in contrast to the results of Yuan’s study, which found that the IPRS method resulted in higher levels of MUFAs, EPA, and DHA [14]. Studies have indicated that the levels of PUFAs, EPA, and DHA rise during low-intensity swimming but decline during high-intensity swimming [23,40]. This explains why the IPRS group, despite exercising, had lower levels of PUFAs, EPA, and DHA, likely due to excessive exercise intensity [41]. The reason why the TPI group had higher contents of PUFAs, Σn-3, EPA, and DHA is that TPI grass carp feed on grass and plankton, with plankton being a major source of Σn-3 polyunsaturated fatty acids like EPA and DHA [42]. The significantly higher ΣSFA content in the IPRS group is similar to findings by Wang and Jia [11,43]. SFAs, as high-energy fatty acids, can supply the necessary energy for swimming in the IPRS group, and being composed of lipid substances, SFAs are closely related to the significantly higher crude fat content in the IPRS group [44]. The IPRS group requires more energy for movement, leading to a relative abundance of energy-related saturated fatty acids; the TPI group, being a static cultivation mode that feeds on natural food like grass, consumes less energy yet achieves a superior muscle nutritional composition compared to IPRS-fed grass carp, aligning with the result of Zhao’s study [45]. This indirectly suggests that fish meat quality post-exercise is not necessarily superior to that of fish without continuous exercise, providing new insights for TPI mode.

Amino acids serve as a crucial metric for assessing the nutritional value of muscle, evaluated through seventeen different types of amino acids, which include nine essential amino acids (EAAs), four non-essential amino acids (NEAAs), and four umami amino acids (UAAs). The presence of EAAs and UAAs is essential for both the nutritional quality and taste of muscle tissue [23]. Our findings showed that the IPRS group exhibited notably elevated amounts of valine and histidine compared to the TPI group (*p* < 0.05), whereas the content of lysine and the total umami amino acids were significantly higher in the TPI group (*p* < 0.05), with no notable differences in ΣEAAs and ΣNEAAs (Table 6). Studies suggest that valine is crucial for muscle production and maintenance, improving the physical flavor characteristics of fish slices and preventing muscle atrophy [46]; histidine is a key element in muscle composition [47]. Research has shown that exercise in fish can enhance muscle characteristics, possibly due to an increase in amino acids that maintain muscle, ensuring quality. Lysine is crucial for the growth of TPI-group grass carp, serving as the primary amino acid that limits their development [48]. Certain research suggests that moderate exercise may boost the levels of EAAs and UAAs in muscle, ultimately improving its taste [49]. This is in opposition to the lower ΣUAA levels in the IPRS group found in this experiment. This discrepancy may result from the exercise intensity in IPRS mode not favoring the synthesis of umami amino acids, or the intercropping aquaculture environment may be better for creating umami amino acids in grass carp muscle. This could be attributed to the grass carp’s natural diet and other natural foods, which may enhance the levels of UAAs in the muscles, as seen in the study by Zhao, who found that grass carp feeding on natural food like grass had higher fatty acids and protein [45]. Glutamate, the primary source of meaty flavor [50], showed no significant difference between the two aquaculture modes (*p* > 0.05), suggesting that pond intercropping leads to an overall increase in UAAs without significantly altering meat flavor and texture. In summary, the TPI-group grass carp had higher ΣUAA content, while the IPRS group had more amino acids that help to generate and maintain muscle tissue, with both aquaculture modes positively impacting muscle quality.

### 4.3. Impact of Different Aquaculture Modes on Volatile Compounds in Grass Carp Muscle

In the IPRS group, the total amount of volatile compounds was higher compared to the TPI group, with 32 types showing significantly higher levels. Conversely, the TPI group exhibited only 12 significantly higher types. The IPRS group exhibited notably elevated levels of nonanal, hexanal, heptanal, hexanol, 1-octen-3-ol, 2-heptanone, 2-butanone, 2-methylbutanal, and 3-methylbutanal compared to the TPI group (*p* < 0.05); conversely, the TPI group had significantly higher ethanol and 2-ethylhexanol contents than the IPRS group (*p* < 0.01) (Table S1). Aldehydes are considered key to fish meat flavor, with nonanal, hexanal, and heptanal identified as markers of fish meat flavor, with nonanal being a primary flavor compound [51,52]. Nonanal has a distinct aroma of fresh green grass and melon [20], while hexanol has a grassy flavor [53]; 1-octen-3-ol is described as having a mushroom-like flavor and a potent vegetal scent [54,55]. 2-Heptanone is considered to have fruity, vanilla, and floral flavors [56]; 2-butanone is associated with a buttery flavor and freshness identification [57,58]. 2-Methylbutanal and 3-methylbutanal are considered to have mushroom and vegetal grass flavors, respectively [59]. Overall, the IPRS group exhibited a variety of flavor aldehydes with intense multiple aromas. Aldehydes are thought to be degradation products of unsaturated fatty acids under the action of enzymes and microbes, closely related to lipid content. Exercise may increase fat content, and in conjunction with the combined action of exercise and lipids, leads to the production of some aromatic aldehydes and other volatile substances to enhance the aroma of fish meat [60]. The TPI group has a high ethanol content, which was different from the results of Ma’s study [12], possibly due to the presence of wild algae and floating plants in the intercropping ponds that can serve as raw materials for ethanol synthesis, resulting in a significantly higher ethanol content in grass carp muscle compared to the IPRS group [61]. The TPI group also exhibited a high content of 2-ethylhexanol, imparting a strong earthy aroma, attributed to the typically sediment-rich environment of TPI ponds, where grass carp inhabit the middle-to-lower layers of water with high soil content, leading to an increase in the volatile compound content imparting an earthy aroma to the muscles. In summary, the volatile aroma of grass carp meat in the IPRS group is primarily influenced and regulated by exercise, with exercise significantly modulating the meat’s aroma, resulting in multidimensional changes in aroma, which is relatively fresh and pleasant. The meat aroma of the TPI group is easily influenced by the environment, characterized by a strong wine aroma but also containing a pronounced earthy aroma, indicating that sediment-rich soil and abundant algae in intercropping ponds have a significant impact on the flavor of the meat.

### 4.4. Impact of Different Aquaculture Modes on Serum Biochemical Indices of Grass Carp

Serum biochemical indicators are closely related to the physiological status of fish, and their changes are accompanied by changes in substances such as proteins, lipids, and sugars in the body. The serum biochemical indicators typically remain within normal range. Abnormal conditions in a fish’s body, including lesions, injuries, or other irregularities, can lead to abnormalities in serum biochemical indicators. The total protein, albumin, and alkaline phosphatase in serum play important roles in fish metabolism and immune function, with albumin being the most abundant protein in plasma, closely related to fish immunity and the health index [62,63]. The findings indicated that the levels of serum total protein and alkaline phosphatase were notably elevated in the IPRS group compared to the TPI group (*p* < 0.01), with the albumin content also being significantly higher in the IPRS group than the TPI group (*p* < 0.05) (Table 7), consistent with the findings of Ma’s study [12]. This is related to the continuous exercise of the IPRS group. According to Zhang’s study, exercise can improve fish health, relying on the proteasome system in the fish body, accelerating protein turnover, regulating the dynamic changes of various enzyme activities in the serum, and enhancing fish metabolism and immune function, specifically reflected in increasing the plasma protein level of the IPRS group [64].

Triglycerides and total cholesterol can reflect the content of blood lipids and the lipid metabolism ability of fish [65]. Research indicates that moderate aerobic exercise can lower levels of TG and TCHO [14]. The results of this experiment are similar to those of Wang’s result [66]. The findings indicated a significant increase in TG levels in the IPRS group compared to the non-exercise TPI group (*p* < 0.01), along with a significant decrease in TCHO levels compared to the TPI group (*p* < 0.01) (Table 7). The continuous exercise of the IPRS group continuously promotes aerobic metabolism in fish, thereby increasing their feed intake and feeding frequency, causing an increase in TG and fat content, and participating in regulating the conversion of TG to SFA in the IPRS group [33,44,67,68]. TG can store energy in fish, and the increase in its content indicates that endogenous fat transport is active [32]. The content of TCHO can reflect the liver metabolic ability, indicating that the liver of grass carp in the IPRS group has a strong ability to metabolize cholesterol, which is related to the regulation of protease activity in the fish body by exercise. By improving the immune health of the fish body, the liver metabolic ability can be improved.

Serum levels of ALT and AST are frequently utilized as biomarkers to assess liver conditions. An increase in ALT and AST indicates varying degrees of damage such as liver and pancreatic dysfunction, necrosis, tissue degeneration, and changes in protein metabolism [65,69,70]. ALT and AST levels were notably reduced in the IPRS group compared to the TPI group (Table 7), aligning with the findings of Kharat and Yuan [14,71]. The experimental findings follow a pattern comparable to Zhang’s result, but the AST content of both groups in this experiment is higher than the normal value, with the TPI group significantly exceeding the standard [72]. Various research has indicated that insufficient oxygen levels in water can harm the liver of grass carp and elevate AST levels. Grass carp ingesting a certain amount of selenium will effectively reduce the content of ALT and AST and alleviate liver damage, and grass carp ingesting enough alanine and glycine will improve the growth performance of grass carp larvae and reduce ALT and AST levels [72,73,74]. The high AST content in the IPRS group may be related to the high-density hypoxic runway aquaculture environment, but due to the fact that consuming selenium-containing artificial feed can alleviate the rise of ALT and AST, the content is lower than that in the TPI group. However, grass carp in the TPI group were not artificially fed, and their food sources lacked sufficient selenium, alanine, and glycine. Furthermore, they usually had high stress levels, which may be important causes of liver damage in the TPI group. Carbohydrates in the blood are an important source of energy and are related to the body’s metabolic levels [75]. Bony fish are usually glucose-intolerant and exhibit persistent high blood sugar levels, so the blood sugar levels in the fish can indirectly reflect their metabolic levels [76]. Both groups exhibited decreased GLC levels, with those of the IPRS group being even lower (Table 7), which was different from the results of Ma’s study and similar to the results of Su’s study [12,77]. The fast glucose clearance rate of grass carp plasma leads to lower GLC levels than other fish, which is related to the strong digestion, gluconeogenesis, and glucose metabolism ability of grass carp. According to West’s study, fish in the swimming exercise group exhibited a glucose metabolism rate three times higher than those in the non-swimming exercise group [78]. The swimming exercise group exhibited increased metabolic activity and improved glucose tolerance. Swimming can lower blood sugar, improve metabolic function, and alleviate the hyperglycemic state caused by glucose formation in the body.

### 4.5. Effects of Different Aquaculture Modes on the Visceral Peritoneum of Grass Carp

The visceral peritoneum is a thin film that covers the abdominal cavity and its internal organs; it can cover most of the organs in the abdominal cavity and has the effect of absorbing impact and protecting the internal organs. It can also secrete mucus to reduce friction between organs, playing a role in lubricating and protecting the internal organs. The visceral peritoneum is not a deposition of pollutants and is rich in a large amount of lipid substances. This study showed that there was a significant difference in the color of the peritoneum between the two aquaculture environments (Figure 7). In the IPRS group, the peritoneum appeared lighter in color compared to the TPI group, where it appeared darker. Moreover, the structure of the visceral peritoneum in the IPRS group was more intact and had a greater extent of coverage (Figure 7). The color of the visceral peritoneum varies depending on species and environment. The peritoneum of grass carp is black, determined by genetic factors and related to melanin deposition [79]. The visceral peritoneum is related to its specific physiological and ecological adaptability, but not directly related to its herbivorous habits. Studies have shown that under certain pathological conditions, pigment cells may increase, causing the color of the visceral peritoneum to darken. Changes in the color of the peritoneum may reflect the health status and pathological changes of fish. The swimming exercise of the IPRS group may regulate the physiological status of the fish while reducing melanin deposition, which is also related to different aquaculture environments. Various research studies may offer varying interpretations, considering the impacts of genetic, physiological, and environmental influences on the peritoneum of grass carp. Due to limited research on the fish visceral peritoneum, its mechanism of action still needs further exploration. If more specific scientific research points out the specific reasons for the color change of the fish peritoneum, more detailed explanations will be provided.

## 5. Conclusions

Overall, there are strengths and weaknesses in the quality of each group of grass carp. The TPI group of grass carp muscles exhibits a high water-holding capacity in terms of physical properties and contains more EPA, DHA, Σn-3, and ΣUAAs, while the content of fat, TG, etc., is relatively low. The grass carp muscles in the IPRS group showed significant advantages in the characteristics of muscle fibers and texture properties, with a significantly lower ash content and high ΣSFA and fat contents; the serum biochemical indicators of this group, such as total protein, albumin, and alkaline phosphatase, are higher, and the visceral peritoneum is lighter in color and more complete in morphology. The two aquaculture modes regulate the quality of grass carp from different perspectives. TPI mainly affects the nutritional composition of muscles, while IPRSs mainly affect the texture of muscles, serum biochemistry, and overall health status. In summary, both of these aquaculture modes have the ability to improve the muscle quality of grass carp to a certain extent. This study provides a reference basis for regulating the nutritional quality of grass carp muscle using TPI and IPRS aquaculture modes.

## Figures and Tables

**Table 1:** Comparison of morphological characteristics of *Ctenopharyngodon idellus* in two aquaculture modes (body length, body weight, condition factor: *n* = 50; the last two indicators: *n* = 9).

Items	TPI	IPRS
Body length/cm	42.53 ± 2.53 **	37.83 ± 2.48
Body weight/kg	1.24 ± 0.21	1.12 ± 0.20
Condition factor/%	1.59 ± 0.47 *	2.05 ± 0.13
Hepatosomatic index/%	1.25 ± 0.14 **	2.15 ± 0.26
Organ Coefficient/%	5.94 ± 0.62 *	6.96 ± 3.68

* indicates significant difference (*p* < 0.05); ** indicates extremely significant difference (*p* < 0.01).

**Table 2:** Comparison of the water-holding capacity of *Ctenopharyngodon idellus* muscles under two aquaculture modes (%, *n* = 3).

Items	TPI	IPRS
Drip loss	7.00 ± 0.95	7.11 ± 0.80
Centrifugal loss	5.53 ± 2.11	4.35 ± 2.01
Liquid loss	14.56 ± 1.97 *	22.54 ± 3.15
Stored loss	1.55 ± 0.84	2.65 ± 0.48
Frozen leakage	2.49 ± 0.47	3.84 ± 0.80
Cooking loss	13.13 ± 1.26 **	26.13 ± 2.51

* indicates significant difference (*p* < 0.05); ** indicates extremely significant difference (*p* < 0.01).

**Table 3:** The long diameter, short diameter, and density of muscle fiber of *Ctenopharyngodon idellus* in two aquaculture modes (*n* = 3).

Aquaculture System	Short Diameter/µm	Long Diameter/µm	Density (n/mm^2^)
IPRS	60.09 ± 6.72 **	114.30 ± 6.50 **	179.24 ± 8.01 **
TPI	84.43 ± 9.68	151.74 ± 13.02	81.32 ± 6.51

** indicates extremely significant difference (*p* < 0.01).

**Table 4:** Analysis of approximate muscle composition of *Ctenopharyngodon idellus* in two aquaculture patterns (%, *n* = 3, wet mass).

Items	TPI	IPRS
Moisture	79.64 ± 1.33	77.16 ± 1.18
Ash	5.14 ± 0.09 **	4.88 ± 0.05
Crude lipid	18.09 ± 0.58 *	19.10 ± 0.15
Crude protein	19.63 ± 1.15	20.27 ± 0.97

* indicates significant difference (*p* < 0.05); ** indicates extremely significant difference (*p* < 0.01).

**Table 5:** Fatty acid contents in muscle of *Ctenopharyngodon idellus* in two aquaculture modes (%, *n* = 3, dry matter).

Fatty Acids	IPRS	TPI
C14:0	0.69 ± 0.10	0.69 ± 0.03
C16:0	27.03 ± 0.50 **	24.31 ± 0.87
C18:0	11.72 ± 0.62	11.07 ± 0.71
C24:0	2.69 ± 0.42 *	1.77 ± 0.35
ΣSFAs	42.13 ± 1.54 *	38.39 ± 1.36
C16:1	1.63 ± 0.23	1.76 ± 0.19
C18:1n-9t	1.52 ± 0.34	1.39 ± 0.05
C18:1n-9c	12.44 ± 0.64	13.31 ± 0.75
C20:1	0.80 ± 0.02	0.76 ± 0.12
C22:1n-9	11.68 ± 0.62	12.11 ± 0.67
C24:1n-9	2.06 ± 0.20 **	0.86 ± 0.37
ΣMUFAs	30.12 ± 0.81	30.20 ± 0.41
C18:2n-6t	1.65 ± 0.23 *	1.26 ± 0.06
C18:2n-6c	8.89 ± 0.53 *	10.59 ± 0.58
C18:3n-3	0.89 ± 0.07 **	1.55 ± 0.18
C20:2	0.91 ± 0.02 **	0.77 ± 0.00
C20:3n-6	1.75 ± 0.13 *	1.44 ± 0.03
C20:4n-6	3.27 ± 0.37	2.96 ± 0.11
C20:5n-3(EPA)	1.41 ± 0.17 **	2.44 ± 0.07
C22:6n-3(DHA)	8.98 ± 0.41 **	10.24 ± 0.10
ΣPUFAs	27.75 ± 0.73 **	31.41 ± 0.95
EPA + DHA	10.39 ± 0.58 **	12.67 ± 0.12
Σn-3	11.28 ± 0.65 **	14.22 ± 0.16
Σn-6	15.55 ± 0.46	16.26 ± 0.58
Σn-9	27.70 ± 0.66	27.68 ± 0.16

ΣSFAs represents the total content of saturated fatty acids. ΣMUFAs represents the total content of monounsaturated fatty acids. ΣPUFAs represents the total content of polyunsaturated fatty acids. Σn-3 represents the total content of ω-3 fatty acids. Σn-6 represents the total content of ω-6 fatty acids. Σn-9 represents the total content of ω-9 fatty acids. The suffix with c is cis fatty acid, the suffix with t is trans fatty acid, and unspecified ones are assumed to be cis fatty acid. * indicates significant difference (*p* < 0.05); ** indicates extremely significant difference (*p* < 0.01).

**Table 6:** Comparative analysis of amino acid composition and content in muscle of *Ctenopharyngodon idellus* in two aquaculture modes (% of total amino acid content, dry matter, *n* = 3).

Amino Acids	TPI	IPRS
Aspartic acid ☆	10.76 ± 0.06	10.80 ± 0.06
Threonine ★	3.95 ± 0.05	3.94 ± 0.04
Serine	3.18 ± 0.06	3.12 ± 0.06
Glutamic acid ☆	16.08 ± 0.15	15.87 ± 0.03
Glycine ☆	4.80 ± 0.21	4.72 ± 0.02
Alanine ☆	6.11 ± 0.06	6.11 ± 0.01
Cysteine	0.90 ± 0.02	0.91 ± 0.04
Valine ★	5.43 ± 0.01 *	5.53 ± 0.05
Methionine ★	3.14 ± 0.03	3.06 ± 0.06
Isoleucine ★	5.17 ± 0.10	5.20 ± 0.04
Leucine ★	8.63 ± 0.03	8.66 ± 0.01
Tyrosine	3.44 ± 0.03	3.43 ± 0.04
Phenylalanine ★	4.95 ± 0.02	4.96 ± 0.02
Lysine ★	10.66 ± 0.26 *	10.42 ± 0.05
Histidine ★	2.95 ± 0.07 *	3.19 ± 0.09
Arginine ★	6.34 ± 0.10	6.46 ± 0.03
Proline	3.52 ± 0.07	3.61 ± 0.04
ΣEAAs	51.22 ± 0.21	51.42 ± 0.03
ΣNEAAs	48.78 ± 0.21	48.58 ± 0.03
ΣUAAs	37.74 ± 0.12 *	37.51 ± 0.07

★ indicates essential amino acids, ☆ indicates umami amino acids, EAAs indicates total content of essential amino acids, NEAAs indicates total content of non-essential amino acids, and UAAs indicates total content of umami amino acids. * indicates significant difference (*p* < 0.05).

**Table 7:** The serum biochemical results of *Ctenopharyngodon idellus* in two modes (*n* = 6).

Items	TPI	IPRS
TP (g/L)	25.39 ± 0.38 **	31.88 ± 0.14
GLC (mmol/L)	7.82 ± 0.06 **	6.61 ± 0.02
ALB (g/L)	13.75 ± 0.11 **	14.72 ± 0.26
TG (mmol/L)	1.48 ± 0.01 **	3.08 ± 0.02
TCHO (mmol/L)	6.05 ± 0.05 **	5.06 ± 0.04
ALT (U/L)	6.29 ± 0.16 **	5.35 ± 0.29
ALP (U/L)	82.68 ± 0.64 **	117.46 ± 0.14
LZM (U/L)	31.5 ± 3.84	34.61 ± 1.07
AST (U/L)	268.54 ± 0.11 **	118.59 ± 0.84

** indicates extremely significant difference (*p* < 0.01).

## Data Availability

The original contributions presented in this study are included in the article; further inquiries can be directed to the corresponding author.

## Supplementary Materials

The following supporting information can be downloaded at https://www.mdpi.com/article/10.3390/foods13081248/s1: Figure S1: Qualitative analysis of volatile substances in *Ctenopharyngodon idellus* muscle in two aquaculture modes. The ordinate represents the retention time of gas chromatography (s), and the abscissa represents the ion migration time (normalized treatment); Table S1: Qualitative results of gas-phase ion mobility spectra of *Ctenopharyngodon idellus* muscle under different aquaculture conditions (*n* = 6).
