# Supplementary material for: Comparative Analysis of Nutritional Quality, Serum Biochemical Indices, and Visceral Peritoneum of Grass Carp (Ctenopharyngodon idellus) Fed with Two Distinct Aquaculture Systems
Source: Foods. 2024 Apr 19;13(8):1248. doi: 10.3390/foods13081248 (PMC11049102; doi:10.3390/foods13081248)
Supplement: Supplementary file 1 [file foods-13-01248-s001.zip › Table S1.pdf]

Table S1. Qualitative results of gas phase ion mobility spectra of *Ctenopharyngodon idellus* muscle under different aquaculture conditions (*n*=6).

Count	Compound	CAS#	Formula	MW	RI	Rt [sec]	Dt [a.u.]	Peak intensity		Comment
								TPI	IPRS	
1	Nonanal-M	C124196	$C_9H_{18}O$	142.2	1101.8	503.541	1.47535	875.28±274.70	1269.59±115.73	Monomer
2	2-ethyl-1-hexanol-M	C104767	$C_8H_{18}O$	130.2	1029	390.54	1.41406	987.80±117.80**	418.18±29.20	Monomer
3	2-ethyl-1-hexanol-D	C104767	$C_8H_{18}O$	130.2	1028.6	390.073	1.8056	252.41±51.35**	58.30±6.80	Dimer
4	Octanal-M	C124130	$C_8H_{16}O$	128.2	1001.6	355.051	1.40726	374.18±147.10*	652.10±46.91	Monomer
5	2-furanmethanol acetate	C623176	$C_7H_8O_3$	140.1	983.4	336.449	1.4186	99.70.±10.03**	568.91±8.37	
6	methyl-5-hepten-2-one	C110930	$C_8H_{14}O$	126.2	984.7	337.606	1.17789	37.50±7.54	31.62±1.11	
7	5-Methylfurfural	C620020	$C_6H_6O_2$	110.1	979.1	332.54	1.13071	151.34±17.86*	105.46±0.00	
8	1-Octen-3-ol-M	C3391864	$C_8H_{16}O$	128.2	976.6	330.242	1.1602	80.33±22.50**	582.46±24.93	Monomer
9	Propyl isovalerate	C557006	$C_8H_{16}O_2$	144.2	945.5	303.344	1.34838	191.50±22.47**	61.24±6.26	
10	2-Octanone	C111137	$C_8H_{16}O$	128.2	990.1	342.656	1.33575	73.85±39.71	44.40±1.48	
11	3-Octanone	C106683	$C_8H_{16}O$	128.2	983.6	336.641	1.30562	28.87±6.93	31.61±6.55	
12	1-heptanol	C111706	$C_7H_{16}O$	116.2	970.4	324.724	1.40456	56.88±22.61	76.42±5.93	
13	Benzaldehyde	C100527	C_7H_6O	106.1	954.3	310.701	1.15318	59.35±15.00**	231.38±11.99	
14	Nonanal-D	C124196	$C_9H_{18}O$	142.2	1101.2	502.415	1.94619	68.81±31.39*	142.71±24.07	Dimer
15	(E)-2-octenal	C2548870	$C_8H_{14}O$	126.2	1054.1	426.359	1.33268	28.53±9.45**	200.70±12.25	
16	benzene acetaldehyde	C122781	C_8H_8O	120.2	1026.1	386.622	1.26488	46.30±3.10**	125.80±9.22	
17	Octanal-D	C124130	$C_8H_{16}O$	128.2	1001.3	354.576	1.82713	53.87±17.60**	182.53±33.82	Dimer
18	(E)-hept-2-enal-M	C18829555	$C_7H_{12}O$	112.2	949.1	306.293	1.25827	53.55±14.45**	369.34±25.96	Monomer
19	(E)-hept-2-enal-D	C18829555	$C_7H_{12}O$	112.2	950.1	307.176	1.67129	12.76±6.09**	73.74±9.12	Dimer

20	1-Octen-3-ol-D	C3391864	$C_8H_{16}O$	128.2	976.6	330.276	1.6026	15.32±9.26**	179.50±23.35	Dimer
21	Heptanal-M	C111717	$C_7H_{14}O$	114.2	890.8	261.245	1.33439	374.37±116.58*	589.61±60.12	Monomer
22	Heptanal-D	C111717	$C_7H_{14}O$	114.2	891.2	261.513	1.69582	73.25±39.03**	531.93±52.54	Dimer
23	2-heptanone-M	C110430	$C_7H_{14}O$	114.2	880.7	255.067	1.26243	182.24±92.93	225.35±11.30	Monomer
24	n-Hexanol-M	C111273	$C_6H_{14}O$	102.2	861.2	243.517	1.32948	499.63±132.02*	906.44±103.53	Monomer
25	n-Hexanol-D	C111273	$C_6H_{14}O$	102.2	859.4	242.442	1.64185	86.07±41.34**	472.77±45.52	Dimer
26	Hexanal-M	C66251	$C_6H_{12}O$	100.2	788.1	204.67	1.26433	812.03±126.24**	1222.90±57.54	Monomer
27	Hexanal-D	C66251	$C_6H_{12}O$	100.2	782.3	201.895	1.55866	899.61±333.41**	4556.41±152.47	Dimer
28	pentan-1-ol-M	C71410	$C_5H_{12}O$	88.1	754	189.564	1.25682	238.98±71.41**	763.48±53.44	Monomer
29	2-Hexanone	C591786	$C_6H_{12}O$	100.2	767.6	195.422	1.18924	122.86±30.18	131.19±10.86	
30	Pentanal-M	C110623	$C_5H_{10}O$	86.1	678.7	160.895	1.19375	419.90±18.63**	840.17±62.03	Monomer
31	2-Pentanone-M	C107879	$C_5H_{10}O$	86.1	665.5	157.049	1.12175	141.73±6.50**	18.70±2.39	Monomer
32	2-Pentanone-D	C107879	$C_5H_{10}O$	86.1	666.8	157.437	1.36984	136.65±10.81*	105.74±10.06	Dimer
33	2-methyl-1-propanol-D	C78831	$C_4H_{10}O$	74.1	604.1	140.34	1.3646	234.62±7.65**	62.07±6.41	Dimer
34	2-butanone-D	C78933	C_4H_8O	72.1	556.8	128.683	1.24492	1034.70±81.76	1217.82±99.34	Dimer
35	Acetoxyacetone	C592201	$C_5H_8O_3$	116.1	515.2	119.228	1.19425	228.53±9.98**	129.38±14.49	
36	Ethanol	C64175	C_2H_6O	46.1	389.1	94.619	1.13223	1478.72±85.66**	48.16±5.23	
37	2-Propanone	C67641	C_3H_6O	58.1	464.3	108.607	1.11738	1858.74±339.45	1135.62±337.14	
38	2-methylbutanal-M	C96173	$C_5H_{10}O$	86.1	642.5	150.572	1.16542	66.25±4.97*	76.82±3.75	Monomer
39	3-methylbutanal-M	C590863	$C_5H_{10}O$	86.1	633	147.982	1.18552	462.56±4.95*	505.53±20.98	Monomer
40	2-methylbutanal-D	C96173	$C_5H_{10}O$	86.1	645.3	151.35	1.38818	42.41±1.76	40.43±6.29	Dimer
41	3-methylbutanal-D	C590863	$C_5H_{10}O$	86.1	634.5	148.371	1.40129	50.44±1.91	49.48±6.95	Dimer

42	2-methylbutan-1-ol	C137326	$C_5H_{12}O$	88.1	719.9	175.7	1.23444	17.24±1.56*	13.12±1.75	
43	3-methylbutan-1-ol	C123513	$C_5H_{12}O$	88.1	719.6	175.57	1.24841	18.08±2.48*	10.58±2.63	
44	2-heptanone-D	C110430	$C_7H_{14}O$	114.2	879	254.012	1.63564	44.81±26.05**	192.53±35.96	Dimer
45	Cyclohexanone	C108941	$C_6H_{10}O$	98.1	886.8	258.765	1.14712	25.22±4.02**	70.71±2.49	
46	(E)-2-hexenal-M	C6728263	$C_6H_{10}O$	98.1	837.7	230.246	1.1818	86.29±13.80**	277.70±6.73	Monomer
47	(E)-2-hexenal-D	C6728263	$C_6H_{10}O$	98.1	837.1	229.929	1.51653	8.36±4.31**	75.65±13.03	Dimer
48	(E)-2-pentenal-M	C1576870	C_5H_8O	84.1	738.3	183.032	1.10792	46.74±8.21**	304.58±14.91	Monomer
49	(E)-2-pentenal-D	C1576870	C_5H_8O	84.1	735.9	182.067	1.36207	9.69±5.76**	177.72±24.76	Dimer
50	Pentanal-D	C110623	$C_5H_{10}O$	86.1	679.2	161.059	1.42377	96.69±14.79**	1741.68±45.54	Dimer
51	Propanoic acid	C79094	$C_3H_6O_2$	74.1	701.5	168.629	1.10679	35.37±4.44**	57.18±3.68	
52	pentan-1-ol-D	C71410	$C_5H_{12}O$	88.1	752.5	188.921	1.51941	63.83±16.58**	856.41±67.64	Dimer
53	2-methyl-1-propanol-M	C78831	$C_4H_{10}O$	74.1	602.7	139.973	1.17507	632.36±6.44**	418.71±29.31	Monomer
54	2-butanone-M	C78933	C_4H_8O	72.1	558.5	129.081	1.0586	706.07±6.03*	913.81±88.89	Monomer
55	pent-1-en-3-ol	C616251	$C_5H_{10}O$	86.1	658.7	155.1	0.94588	135.76±18.95**	826.70±28.53	

* indicates a significant difference (p < 0.05), ** indicates a highly significant difference (p < 0.01). RI is retention index, Rt is retention time, Dt is migration time, [RIP rel] means normalization.
