# Supplementary figures and images for: Comparative Analysis of Nutritional Quality, Serum Biochemical Indices, and Visceral Peritoneum of Grass Carp (Ctenopharyngodon idellus) Fed with Two Distinct Aquaculture Systems
Source: Foods. 2024 Apr 19;13(8):1248. doi: 10.3390/foods13081248 (PMC11049102; doi:10.3390/foods13081248)

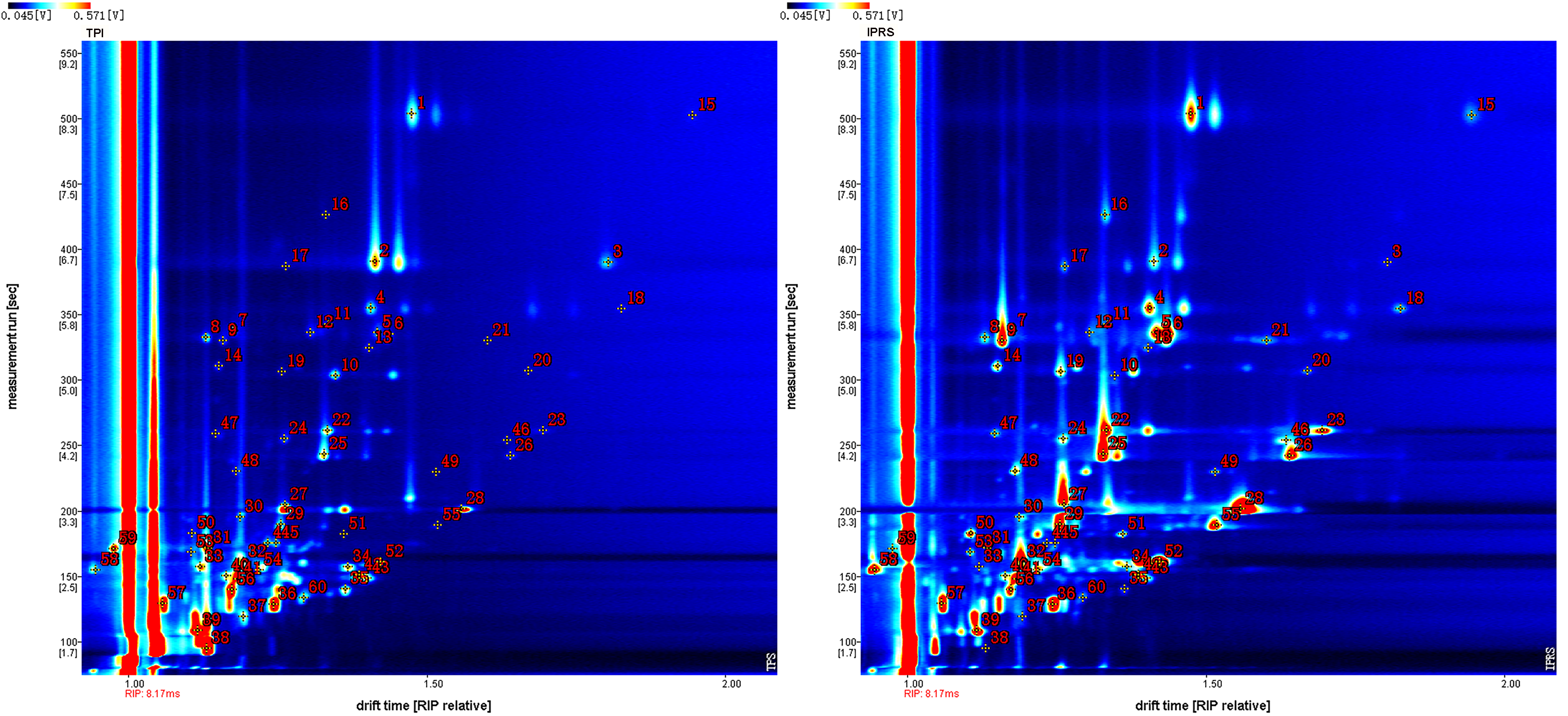

Supplement: Supplementary file 1 [file foods-13-01248-s001.zip › Figure S1.tif]
